# CD5L is a potential negative regulator of chondrocyte apoptosis in osteoarthritis

**DOI:** 10.1016/j.ocarto.2026.100849

**Published:** 2026-07-04

**Authors:** Benjamin Brigant, Kashif Rasheed, Carolina Gómez Ortega, Erlend Tande, Shiquan Sun, Xuzhao Bian, Victor Boyartchuk

**Affiliations:** a Department of Clinical Research and Molecular Medicine (IKOM), Faculty of Medicine and Health Sciences (MH), Norwegian University of Science and Technology (NTNU), Trondheim, Norway; b School of Public Health, Xi’an Jiaotong University, Xi’an, Shaanxi, 710061, PR China; c Surgery Clinic, St. Olav’s Hospital HF, Trondheim, Norway; d Centre for Integrative Genetics, Department of Animal and Aquacultural Sciences, Faculty of Biosciences, Norwegian University of Life Sciences, Ås, Norway

**Keywords:** CD5L, Doxorubicin, Osteoarthritis, IL1β, Chondrocyte

## Abstract

**Objectives:**

CD5 antigen-like (CD5L) protein is a scavenger receptor involved in inflammation control and enriched in the synovial fluid and membranes of osteoarthritis (OA) patients. Although increased CD5L levels have been associated with OA pathology, its direct role in chondrocytes and contribution to cartilage degeneration remain unknown. We investigated the impact of CD5L on human chondrocytes in the context of OA.

**Methods:**

We compared CD5L mRNA levels in primary chondrocytes between OA patients and unaffected controls. We also measured CD5L levels in two chondrocyte cell lines under conditions mimicking OA. RNAseq was performed to determine the effects of increasing CD5L expression on the chondrocyte transcriptome. Finally, we studied the influence of CD5L levels on chondrocyte survival by depleting or overexpressing CD5L in chondrocyte cell lines.

**Results:**

CD5L transcript levels were higher in primary chondrocytes from OA patients than in those from controls. Treatment with the pro-inflammatory cytokine IL1β or doxorubicin further induced CD5L expression in primary and cultured chondrocytes. Transcriptomic analysis revealed a correlation between high CD5L transcript levels and expression of genes involved in programmed cell death, inflammation, and differentiation. CD5L knockout increased survival of doxorubicin-treated chondrocytes and reduced apoptosis marker levels compared to non-targeting controls.

**Conclusion:**

Our data indicate that CD5L plays an important role in regulating chondrocyte cell death. CD5L expression levels are high in chondrocytes from OA patients, and decreasing CD5L expression reduces chondrocyte apoptosis induction *in vitro*. CD5L is therefore a potential target for OA management.

## Graphical abstract

**Figure:**
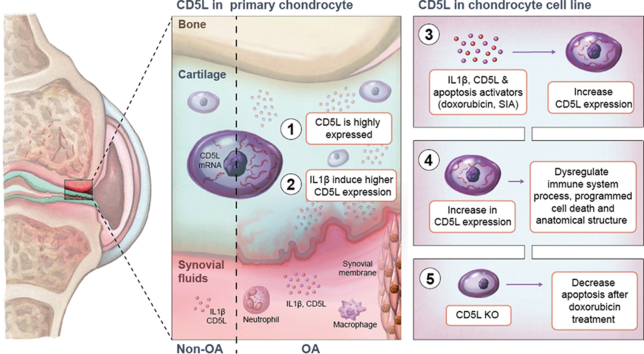

## 1 Introduction

Osteoarthritis (OA) is one of the most frequent human chronic diseases and the single most frequent cause of disability in older adults. It is a highly prevalent, high-burden chronic disease with progressive increases in incidence and risk factors every year [1]. Current therapeutic options for OA patients are limited to pain management [2] and, in cases of severe symptoms, joint replacement surgery [3]. There is, therefore, an urgent need to identify new therapeutic targets for halting OA progression.

OA is a chronic joint disease characterized by systemic dysfunction and degradation of the articular cartilage. Chondrocytes are the only cell type present in the articular cartilage. These specialized cells are responsible for maintaining the balance of joint physiology and the composition of the extracellular matrix (ECM) [4]. Cartilage deterioration during OA progression is a multifaceted process. It involves chondrocyte cell death, complex inflammatory responses, and the degradation of ECM components, but the molecular mechanisms underlying cartilage breakdown remain poorly understood.

CD5L (also known as Spα or *AIM*) was originally identified as a molecule promoting the survival of immune cells in mice [5]. It is a secreted multifunctional scavenger receptor-like protein that is abundant in blood and acts by binding and sequestering a wide range of molecules, including immunoglobulin M [6], oxidized lipids [7] and apoptotic cell fragments via KIM-1 interaction [8], and. CD5L also has an important intracellular function, regulating the abundance of intracellular lipids that serve as ligands for nuclear receptor transcription factors [9].

CD5L accumulation has been reported in serum, synovial fluid (SF), and synovial membranes of rheumatoid arthritis (RA) and OA patients. Balakrishnan. et al. showed that in OA patients, SF CD5L protein levels are more than three times higher than those in RA patients [10]. This observation is further supported by Wu et al. who demonstrated that serum CD5L concentration is elevated in patients with OA (x1.6) or RA (x3.6) when compared to healthy controls [11]. The same team recently detected high levels of CD5L in the synovial membranes of both RA patients and rats with collagen-induced arthritis. Remarkably knocking down expression of the CD5L receptor CD36 in rat models mitigated the harmful effects of exogenous CD5L treatment [12]. Obesity is a major risk factor in OA [13] and Shoji et al. recently showed that CD5L is overexpressed in the synovial membranes of obese patients with OA. However, no study to date has directly queried CD5L function in human chondrocytes—the principal cell type of cartilage, and a key player in OA pathogenesis.

In this study, we fill this critical gap by, for the first time, systematically evaluating CD5L expression and function in primary human chondrocytes from OA and non-OA patients, as well as in two human chondrocyte cell lines (TC28A2 and CHON002). We employed both genetic loss-of-function (CRISPR knockout (KO)) and gain-of-function strategies, under pro-apoptotic and inflammatory conditions, to uncover the mechanistic contribution of CD5L to chondrocyte survival and apoptotic responses.

IL1β is a pro-inflammatory cytokine commonly found at elevated levels in the SF of OA patients [14]. It is frequently used *in vitro* to induce pro-catabolic and inflammatory responses in chondrocytes [15]. This cytokine reduces the ability of chondrocytes to generate ECM and amplifies their catabolic actions, leading to ECM degradation. Doxorubicin is a chemotherapy agent used to treat human cancer that can induce the apoptosis of chondrocytes and promote cellular senescence *in vitro* [[16], [17], [18]] and *in vivo* [19]. Doxorubicin also increases the expression of matrix-degrading enzymes, such as MMP-13 [20]. We used these OA-inducing agents to (i) validate the induction of CD5L expression in an OA environment in chondrocyte cell lines and primary samples, (ii) study the effect of CD5L KO on resistance to cell death after doxorubicin treatment, and (iii) investigate the effect of CD5L on gene expression in the CHON002 cell line.

## 2 Materials and methods

### 2.1 Human primary chondrocytes

Primary chondrocytes were isolated from the knee joints of three OA patients or from the vertebral transverse costal facet joints of three patients without OA (non-OA controls), as previously described [15]. Non-OA controls (NCs) were patients who underwent spinal surgery for non-arthritic and non-inflammatory indications (e.g., scoliosis correction). All patients gave informed consent, and chondrocyte collection was approved by the French Ministry of Higher Education and Research (registration number: DC-2017–2987). Chondrocytes were cultured for 24 h in the presence of complete medium with or without IL1β (at a concentration of 1 or 10 ng/mL) before total RNA extraction.

### 2.2 Publicly available transcriptomic data

To validate CD5L expression changes in a larger independent cohort, we retrieved and analyzed bulk RNA-seq data from the publicly available dataset GSE114007 (NCBI Gene Expression Omnibus), comprising 18 normal and 20 OA human knee cartilage tissue samples [21]. As previously described [22], we performed deconvolution analysis using CIBERSORTx (https://cibersortx.stanford.edu/) to integrate scRNA-seq data with bulk RNA-seq data. A signature matrix was constructed from marker genes of chondrocyte populations identified in the scRNA-seq dataset. The relative proportions of chondrocyte populations in bulk RNA-seq samples were then estimated by CIBERSORTx. Based on the inferred cell-type proportions, OA bulk samples were classified into Group A and Group B using hierarchical clustering. Group A represented an inflammatory/immune-related subtype, whereas Group B represented an ECM-related subtype, consistent with previous reports. Normalized CD5L expression was visualized in OA versus normal samples and across OA subgroups versus normal samples. Group differences were assessed using the Wilcoxon rank-sum test.

### 2.3 Cell lines

The CHON-002 human chondrocyte cell line is derived from the long bone cartilage of an 18-week-old female fetus. This cell line (CHON-002 ATCC ® CRL-2847™) was purchased from the American Type Culture Collection (Manassas, VA, USA). It was cultured in Dulbecco’s Modified Eagle Medium (BE12-604F, Lonza) supplemented with 0.1 mg/mL G-418, 10% fetal bovine serum (10270, Gibco) and penicillin-streptomycin antibiotics (P0781, Sigma-Aldrich), in a humidified atmosphere containing 5% CO_2_, at 37 °C.

The TC28a2 human chondrocyte cell line was obtained from the CellCOM Research Group (Universidade da Coruña, Spain) and was used for independent confirmation of the key results of the study. The TC28a2 cell line was cultured in similar conditions to the CHON002 cell line but without G-418.

For lentivirus production, the HEK293T cell line was cultured under the same conditions as TC28a2 cells.

Dulbecco’s Phosphate-Buffered Saline (D8537, Sigma-Aldrich) was used to wash the cells during maintenance, and 0.25% trypsin-EDTA (BE17-161E, BioWhittaker) (170,000 U/L trypsin 1:250 and 0.2 g/L EDTA) was used to detach the cells after confluence was reached. For short-term storage the cells were frozen in 90% FBS/10% DMSO at in liquid nitrogen.

### 2.4 Cell treatment

To induce apoptosis cells were exposed to doxorubicin (#44583, Sigma Aldrich, US) or sodium iodoacetate (#I2512, Sigma Aldrich, US). For validation of the effect of inflammation and CD5L on chondrocytes, we used recombinant IL1β (200-01B, PeproTech) and recombinant CD5L (10791-H08H, Nordic Biosite) at the concentrations and for the times indicated in the legends to the figures. Recombinant proteins were heated at 95 °C for 30 min to create denatured controls.

### 2.5 Generation of CD5L KO cell lines

The KO cell lines were generated by lentiviral transduction of constructs carrying individual sgRNA guides inserted into the lentiCRISPRv2-Opti vector (Addgene # 163126) according to the Addgene cloning protocol, Full details of the CRISPR-Cas9 protocol, including sgRNA design, oligonucleotide cloning, lentiviral production, transduction conditions, puromycin selection, and KO validation, are provided in the Supplementary Methods.

### 2.6 Generation of CD5L overexpression (OE) cell lines

The OE line was generated by lentiviral transduction of both CHON-002 and TC28A2 cell line with the PLJM1 plasmid containing the CD5L cDNA under the control of the CMV promoter and a puromycin resistance gene (Addgene #91980).

### 2.7 Reverse transcription and quantitative PCR

Total RNA was isolated from chondrocytes using the RNeasy Plus Mini Kit (#74034, Qiagen) according to the manufacturer’s instructions. RNA quality was verified by NanoDrop spectrophotometry (OD260/OD280 ≈ 2). Equal amounts of total RNA were reverse-transcribed into cDNA using either the High-Capacity RNA-to-cDNA Kit (Applied Biosystems) or the Verso™ cDNA Synthesis Kit (Thermo Fisher Scientific, AB-1453/B). QPCR was performed in 20 μL reactions containing 100 ng cDNA and SYBR Green Master Mix on an ABI StepOnePlus Real-Time PCR System. Relative gene expression levels were determined using the comparative CT (ΔΔCT) method with GAPDH as an endogenous control. Primer sequences (Table 2) are provided in the supplementary material.

### 2.8 RNAseq analysis

Total RNA was isolated from CHON-002 cell line overexpressing CD5L and the control line transduced with empty vector, as described above. RNA library preparation and transcriptome sequencing was performed by Novogene Europe (Cambridge Science Park, United Kingdom). Libraries were sequenced on the Illumina NovaSeq 6000 system to generate 6 GB of 150-base paired-end reads. Short sequence reads were pseudo-aligned with the GRCh38 v110 human transcriptome using the *kallisto* [23] read-alignment tool. For each alignment, 50 bootstraps were generated to model experimental variability. Transcript quantification, normalization and gene aggregation was performed with the *sleuth* R package [24]. The resulting tpm values were used to identify genes differentially expressed (DE) as a result of CD5L OE. Annotation and enrichment analysis were performed on the pool of potential DE genes with *shinyGO* v 0.77 tool [25]. A list of genes detected at >0.1 tpm was used as the background for enrichment analysis.

### 2.9 Western-blot analysis

Cells were lysed in RIPA buffer (Sigma Aldrich, MS, USA) in the presence of protease inhibitor cocktail (Sigma Aldrich - P8340 – 1/100). Lysates were centrifuged at 12,000×*g* and 4 °C for 15 min and supernatants were collected for protein quantification and Western-blot analysis. Equal amounts of protein (40 μg) were mixed in 1X LDS Sample Buffer (NuPage®, Invitrogen) with 50 mM dithiothreitol (DTT), heated for 10 min at 95 °C, sonicated for 1 min and centrifuged for 10 min at 10000 g Protein extracts were separated by electrophoresis in 4–12% Bis-Tris mini protein gels (NuPage®, Invitrogen) and the resulting bands were transferred onto nitrocellulose membrane. Membranes were blocked for 1 h with 5% non-fat dry milk in Tris-Buffered Saline supplemented with 0.01% Tween 20 (TBST). The primary antibodies against CD5L (mouse monoclonal F1 clone, 1/1000, sc-514283, Santa Cruz Biotechnology) and GAPDH (1/2000, sc-47724, Santa Cruz Biotechnology) and the HRP-conjugated goat anti-mouse secondary antibody (1/5000, #P0447, Dako) were diluted in 0.5 X blocking buffer/PBS. The bound HRP-conjugated secondary antibodies were detected with the SuperSignal TM West Femto Maximum Sensitivity Substrate (Thermo Scientific). Chemiluminescent signals were captured with an Odyssey® Fc Imager (926–40020, Li-Cor Biosciences).

### 2.10 Viability assay

The CellTiter-Glo Luminescent Cell Viability assay (#G7571, Promega) was used to assess cell proliferation in accordance with the manufacturer’s instructions. In brief, CHON002 cells (1.0 × 10^3^ per well in 12-well plates) or TC28A2 cells (1.0 × 10^3^ per well in 12-well plates) were cultured for 5 h (for D0), and then for 24, 48 or 72 h at 37 °C. We then mixed 100 μL CellTiter-Glo solution (Promega) and 100 μL H_2_0 directly with the culture medium and incubated the cells for 10 min at room temperature. The mixture was then transferred, in duplicate, to white 96-well plates for the measurement of luminescence intensity. Experiments were performed at least three times.

### 2.11 Immunochemistry

Cartilage/subchondral bone samples were obtained from patients undergoing total knee replacement surgery. They were fixed by incubation for one week in 4% paraformaldehyde and were then placed in 0.5 M EDTA pH8 at 4 °C until complete decalcification was achieved. The samples were embedded in paraffin and 4 μm sections cut and stained with Safranin-O Fast Green according to standard histological procedures. Sections were also stained by IHC with antibodies specific for CD5L overnight at 4 °C (sc-514283; concentration used, 1 μg/mL; Santa Cruz Biotechnology) after proteinase K (Sigma Aldrich, 20 μg/mL) antigen retrieval for 30 min at 37 °C. The primary antibody was detected with the mouse ImmPRESS kit (Vector Laboratories) and the DAB substrate (Agilent).

### 2.12 Immunofluorescence

We used chambered slides (IBIDI, 8 well, #80841) to culture chondrocytes for 24 h before treatment. After additional 24 h of treatment, the cells were washed with PBS followed by blocking solution, consisting of PBS supplemented with 20% FBS and 0.05% saponin to facilitate cell permeabilization prior to incubation with primary antibodies. Unbound antibodies were removed by washing with a buffer consisting of PBS, 1% FBS, and 0.05% saponin. Following 3 washes cells were incubated with secondary antibodies conjugated with fluorophores binding specifically to the primary antibodies (see details Supplementary Material Table 3). DAPI was included during incubation with the secondary antibody, to stain DNA and visualize the nucleus. We used EVOS FL Auto for visualization of the levels, distribution and interactions of proteins of interest in our biological samples.

### 2.13 Apoptosis detection

Apoptosis and necrosis were assessed following doxorubicin treatment using two complementary Annexin V-based approaches: a kinetic bioluminescence assay (RealTime-Glo Annexin V Apoptosis and Necrosis Assay, Promega) and endpoint fluorescence microscopy (Annexin V-FLUOS Staining Kit, Roche; EVOS FL Auto). Full protocols are provided in the Supplementary Methods.

### 2.14 Statistical analysis

GraphPad Prism software (GraphPad Prism Version10.0; GraphPad Software, Inc., San Diego, CA) was used for statistical analysis. We first assessed the normality of the data with the Shapiro-Wilk normality test. Normally distributed continuous variables are presented as means ± standard deviation and were analyzed in one-sample *t*-tests. If the data were not normally distributed, a non-parametric test, such as the Wilcoxon rank-sum test, was used instead. The *p*-value was calculated and considered statistically significant if it was less than 0.05.

## 3 Results

### 3.1 CD5L transcript and protein levels are elevated in tissues of OA patients

**Fig. 1:**
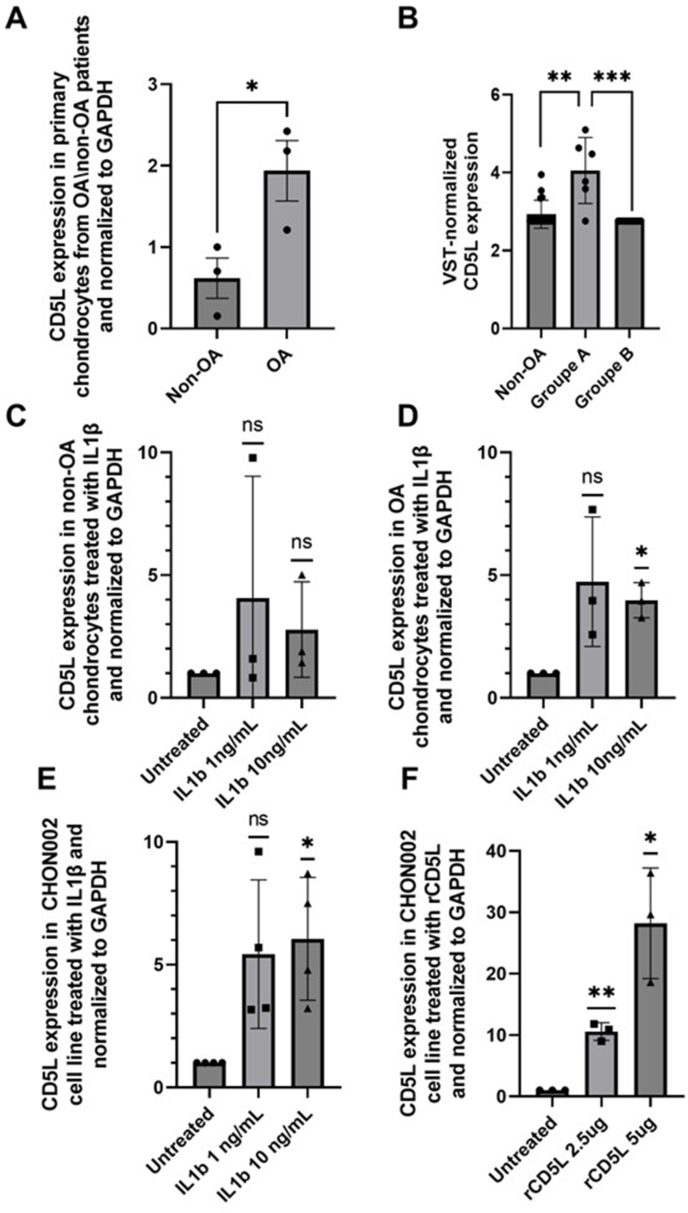
CD5L levels are high in chondrocytes from OA patients and following treatment with IL1β and rCD5L. **A.** CD5L expression using qPCR in primary chondrocytes from OA patients and non-OA patients. **B**. CD5L transcript levels in cartilage from OA patients in subgroups A and B, and from non-OA patients, based on bulk RNA-seq data (GSE114007). Subgroup A corresponds to an inflammatory/immune-related OA subtype, whereas subgroup B corresponds to an ECM-related OA subtype. **C** – **D** CD5L expression in primary chondrocytes from non-OA patients (**C**) or OA patients (**D**) incubated with 1 or 10 ng/mL rIL1β (24 h); **E** CD5L levels in CHON-002 cells after 24 h of treatment with IL1β (*n* = 4); **F** CD5L levels in CHON-002 cells after 24 h of treatment rCD5L (*n* = 3). GAPDH was used for normalization in all RT-qPCR experiments.

To determine if CD5L is DE in tissues impacted by OA we measured production of endogenous CD5L transcript in chondrocytes isolated from patients diagnosed with OA and NCs. To this end we used qPCR to amplify CD5L and GAPDH endogenous control amplicons using cDNA samples generated from total RNA isolated from OA and control patient tissues. A comparison of normalized values for CD5L expression between three OA and three non-OA chondrocyte samples revealed that CD5L mRNA levels were significantly higher in the chondrocytes of OA patients (x1.93, *p* = 0.041; Fig. 1A). Subsequently we confirmed that CD5L protein was present in osteoarthritic cartilage by immunohistochemistry. Almost no CD5L protein was detected in the subchondral bone (Supplementary Fig. 1). To complement our primary cohort qPCR data, we analyzed CD5L expression in the publicly available bulk RNA-seq dataset GSE114007, comprising 18 non-OA and 20 OA human knee cartilage samples. Due to the low basal expression of CD5L, transcript levels were undetectable in a substantial proportion of samples, precluding a straightforward comparison between the overall OA and non-OA groups (Supplementary Fig. 2). However, when OA samples were stratified into two subtypes by hierarchical clustering — subgroup A (inflammatory/immune-related) and subgroup B (ECM-related) — CD5L expression was significantly elevated in subgroup A relative to non-OA samples (x1.38, *p* = 0.0067; Fig. 1B).

### 3.2 IL1β induces CD5L expression in chondrocytes

IL1β is a pro-inflammatory cytokine that has been implicated in the development and progression of OA [26]. It is widely used *in vitro* to induce inflammation in OA studies. We therefore tested the involvement of IL1β in upregulating CD5L in OA chondrocytes. Chondrocytes were treated with 1–10 ng/mL IL1β for 24 h. We found that at the high end of the tested range IL1β treatment significantly increased CD5L levels in the chondrocytes of OA patients (x3.97 with 10 ng/mL; *p* = 0.018; Fig. 1D) and in the CHON002 chondrocyte cell line (x6; *p* = 0.027; Fig. 1C). Interestingly, CD5L expression in non-OA primary chondrocytes was not significantly modified by IL1β exposure (Fig. 1B).

CD5L protein levels in SF from the joints of OA patients have been reported to be more than three times higher than those in healthy controls [11]. We therefore chose to test the effect of exposing our chondrocyte cell lines to exogenous recombinant CD5L. We found that extracellular CD5L enhanced the endogenous expression of CD5L in chondrocytes in a dose-dependent manner (Fig. 1E). CD5L therefore appears to be involved in the regulation of its own expression in chondrocytes, and pro-inflammatory cytokines, such as IL1β, may contribute to the increase in CD5L levels in OA.

### 3.3 Chondrocyte apoptosis induces CD5L expression *in vitro*

CD5L has been earlier implicated in control of cell death we hypothesized that doxorubicin treatment will change the levels if its expression. To test this, we exposed two different chondrocyte cell lines to various amounts of doxorubicin.

**Fig. 2:**
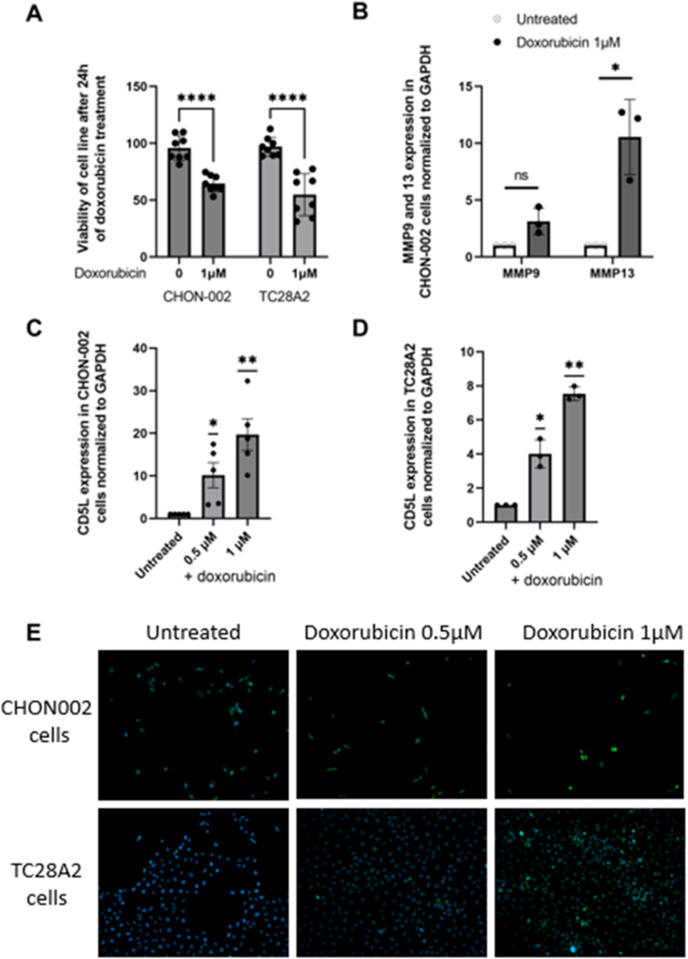
Doxorubicin treatment increases CD5L expression in chondrocyte cell lines. **A.** Viability of chondrocyte cell lines after 24 h of treatment with 1 μM doxorubicin as measured with CellTiter-Glo assays. **B.** MMP9 and MMP13 mRNA levels in the CHON-002 cell line after 24 h of treatment with 1 μM doxorubicin. CD5L levels in the CHON002 (**C**) and TC28A2 (**D**) cell lines after treatment with doxorubicin for 24 h. **E.** Immunofluorescence of CD5L in CHON002 and TC28A2 cell lines treated with doxorubicin for 24 h.

As expected, doxorubicin induced cell death in both chondrocyte cell lines (Fig. 2A). After 24 h of treatment with 1 μM doxorubicin, we observed a decrease in cell viability by about 50% in both cell lines. Our experiments using CHON002 confirmed that doxorubicin treatment increases MMP13 expression in this cell line. In contrast to the pronounced upregulation of MMP13, MMP9—another key enzyme in cartilage degradation—exhibited only a modest, non-significant increase following doxorubicin treatment.

Quantification of mRNA levels from treated and control cells by qPCR revealed a significant and dose-dependent increase in CD5L expression after doxorubicin treatment in both chondrocyte cell lines: CHON002 (10 μM, *p* = 0.073, *n* = 5; Fig. 2C) and TC28A2 (10 μM, *p* = 0.0012, *n* = 3; Fig. 2D). Consistent with changes in CD5L mRNA levels as measured qPCR, we observed an increase in CD5L protein levels by immunofluorescence. CD5L protein was found to have notably elevated in both cell lines after 24 h of doxorubicin treatment (Fig. 2D).

To establish if induction of CD5L expression is specific to doxorubicin or is also present under alternative apoptosis inducing conditions., we also investigated the effects of sodium iodoacetate [27], on changes in the expression of CD5L in the CHON002 cell line. Similarly, to doxorubicin, CD5L transcript levels were significantly higher in iodoacetate treated cells when compared to untreated cells (*n* = 3, *p* = 0.0023) (Supplementary Fig. 2). These findings suggest that induction of CD5L expression in chondrocytes is a general response to cytotoxic treatments.

### 3.4 Effect of CD5L expression levels on cell death and apoptosis

**Fig. 3:**
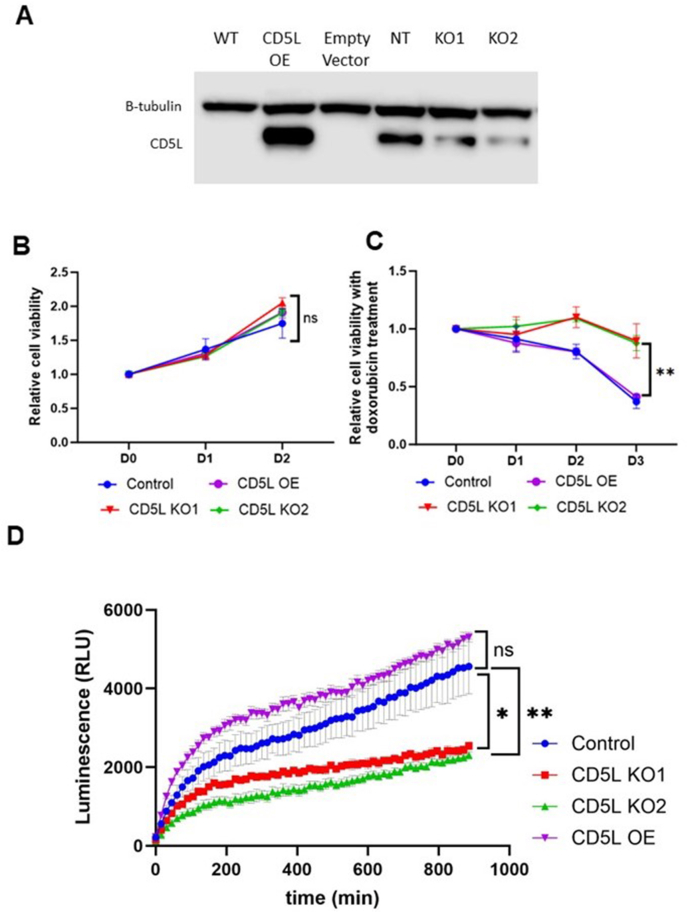
CD5L KO protects chondrocytes against the cell death induced by doxorubicin. **A.** Western blot of CHON002 cells in which CD5L was overexpressed or KO. (**B** and **C**) CellTiter-Glo Luminescent Cell Viability Assay on the CHON002 cell lines grown in the absence (**B**) or presence (**C**) of doxorubicin treatment. Cells were treated with 1 μM doxorubicin of) for 72 h and cell viability was assessed with CellTiter-Glo assay. **D**. RealTime-Glo Annexin V apoptosis assay showing luminescence over 15 h in CHON-002 cells treated with 1 μM doxorubicin, reflecting the time-dependent activation of apoptosis. Error bars indicate the mean ± SEM of at three four independent experiments. ns non-significant, ∗*p* < 0,05, ∗∗*p* < 0.01; ∗∗∗∗*p* < 0.0001.

Doxorubicin is known to induce apoptosis in chondrocytes [19]. To investigate the impact of changes in CD5L levels on chondrocyte viability we generated a series of cell lines in which CD5L was either overexpressed or KO. Due to the low basal expression of CD5L we used an indirect approach to test *CD5L* KO efficiency by western blotting. In such test we overexpressed *CD5L* mRNA before introducing *CD5L* targeting guides to visually monitor depletion of both endogenous and transgenic CD5L protein. Our WB confirmed high efficiency of both guides targeting *CD5L* (Fig. 3A). In subsequent experiments, we utilized separate OE and KO cell lines generated without overexpressing *CD5L* prior to guide transduction.

To confirm that our genetic manipulations themselves did not impact cellular viability or proliferation, we monitored *CD5L* deletion or OE cell lines under normal culture conditions, by using the CellTiter-Glo Luminescent Cell Viability assay for a period of 48 h. We observed no differences in viability between Non-Targeting (NT) guide transduced control cells, KO1, KO2, OE vector control (EV) and OE cells in the absence of OA inducing treatments. Based on these observations, we conclude that changes in CD5L levels do not impact cell proliferation under normal conditions (Fig. 3B).

Subsequently we monitored the viability of doxorubicin-treated chondrocytes with altered CD5L levels in a 72-h time-course experiment. We observed a marked decrease in cell viability in control and OE CHON002 cell lines after doxorubicin treatment (Fig. 3C). CD5L KO cell lines had significantly more live cells, even at the 72 h timepoint. Thus, in the absence of CD5L, chondrocytes are more resistant to cell death induced by doxorubicin treatment. We confirmed that the observed effect was not cell line-specific by replicating the effect of CD5L levels in TC28A2 chondrocyte cell line (Suppl. Fig. 2).

We first monitored apoptosis and necrosis kinetics in CHON-002 cells treated with 1 μM doxorubicin using the RealTime-Glo Annexin V Apoptosis and Necrosis assay. Luminescence measurements revealed a higher apoptotic signal over time in WT and CD5L-overexpressing cells compared with CD5L KO cells (Fig. 3D). In contrast, no appreciable increase in the necrosis-associated fluorescence signal was detected over the 15-h recording period (Suppl. Fig. 5), indicating that cell death under these conditions was predominantly apoptotic. We then confirmed these findings by endpoint Annexin V staining and fluorescence microscopy after 24 h of doxorubicin treatment (1 μM). Consistent with the time-course data, CD5L KO cells displayed a lower percentage of Annexin V–positive cells than WT and CD5L-overexpressing cells (Suppl. Fig. 6A and B). Together, these observations indicate that CD5L promotes doxorubicin-induced apoptosis in chondrocytes.

### 3.5 CD5L OE drives expression of genes linked to programmed cell death

**Fig. 4:**
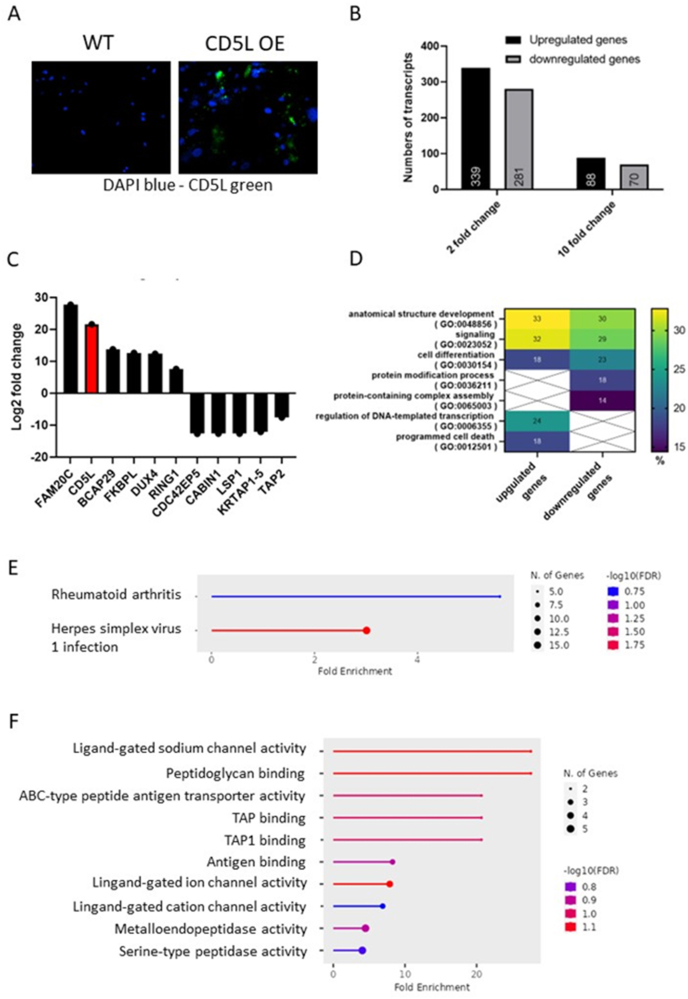
Gene expression in chondrocytes overexpressing CD5L. **A.** Immunofluorescence imaging of CD5L OE in the CHON002 cell line used for RNAseq analysis. CD5L in green (AF-488), nuclear DNA stained with DAPI in blue. **B.** 5.25% of the more than 11800 transcripts detected had a fold-change in tpm of more than 2 and 1.25% had a fold-change in tpm of more than 10; **C**. Top 5 up- and downregulated genes identified by RNAseq analysis sorted by fold-change in tpm. **D.** Heatmap representing the percentage of the top 5 GO terms displaying enrichment among up- and downregulated benes with a fold-change >2. Data from the Generic GO Term Mapper (https://go.princeton.edu/). **E.** Jensen-associated diseases **F**. GO analysis.

We used the CD5L-overexpressing CHON-002 cell line to characterize the global changes in the chondrocyte transcriptome induced by high levels of CD5L. To achieve this, we sequenced mRNA isolated from the control cell population transduced with empty vector and cells stably overexpressing CD5L. CD5L OE was validated by western blotting (Fig. 3A) and by immunofluorescence analysis (Fig. 4A) before RNAseq analysis.

In total, our RNAseq analysis detected 11800 tpm values above 1. 620 transcripts (5.25%) had *tpm* values that differed more than 2-fold between the test and control samples. 148 transcripts (1.25%) had more than 5-fold difference in counts between OE and control samples. Overall, 339 genes were found to be upregulated and 281 were downregulated (fold-change>2, Fig. 4B).

Among the top 10 dysregulated genes in cells overexpressing CD5L were *CABIN1, DUX4*, and *LSP1* (Fig. 4C), all of which have documented involvement in apoptosis [[28], [29], [30], [31], [32], [33]]. *FAM20c* (log_2_ fold-change: 27.74) and *DUX4* (log_2_ fold-change: 12.37) were upregulated and *LSP1* and *CABIN1* were downregulated (log_2_ fold-change: 12.58 and −12.60, respectively) in CD5L-overexpressing cells relative to wild-type cells.

To identify general themes of changes induced by OE of CD5L we tested for enrichment of specific gene sets or functions by performing Gene Ontology (GO) term enrichment analysis using public databases. We used Generic GO Term Finder [34] to identify enrichment independently for the upregulated and downregulated groups of genes. The top three GO terms were the same in both the upregulated and downregulated gene groups. In both groups, anatomical structure development was the most enriched GO term. In the group of downregulated genes, enrichment was also observed for protein modification processes and protein-containing complex assembly. Remarkably, 15% of the genes induced by CD5L OE were associated with the GO term “programmed cell death”, suggesting that CD5L OE leads to the upregulation of genes involved in induction and execution of cell death (Fig. 4D).

Subsequently we explored the extent to which the genes dysregulated by CD5L OE were associated with gene signatures of human diseases, using ShinyGO, a graphical gene-set enrichment analysis tool that integrates access to the majority of public gene-set databases [25]. For the gene-set enrichment analysis, we identified enriched GO terms using a fold-change cutoff of 2 (Fig. 4F) and gene sets linked to diseases (Fig. 4E). DE genes displayed an almost five-fold enrichment in transcripts associated with RA. This pattern of enrichment suggests a connection between CD5L dysregulation and the disruption of transcriptional activity in chondrocytes (Fig. 4E). Furthermore, our data revealed a potential association between CD5L levels, and the dysregulation of genes involved in ligand-gated ion-channel activity and peptidoglycan/TAP binding, highlighting a potential, previously unsuspected role for CD5L in chondrocytes.

Our RNAseq data (Fig. 4) identified several upregulated genes associated with apoptosis. We therefore tested if expression of the genes dysregulated by the OE of CD5L is also altered in primary cells. We used qPCR to analyze the levels of mRNA for a subset of genes in chondrocyte samples from OA and non-OA patients. We focused on three genes, *DUX4, LSP1*, and *CABIN1*, based on published descriptions of their links to apoptosis and the magnitude of changes in expression levels that were induced by CD5L OE (Fig. 4C).

**Fig. 5:**
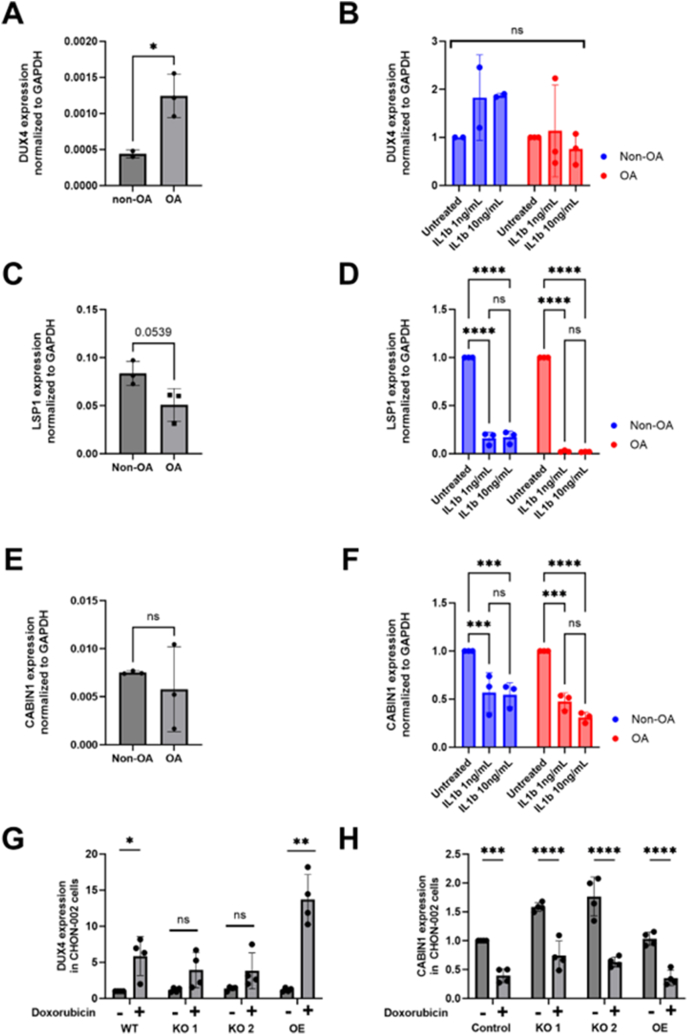
RT-qPCR analysis of candidate gene expression in OA and non-OA primary chondrocytes. *DUX4* (**A-B**), *LSP1*(**C-D**), and *CABIN1* (**E-F**) expression levels in untreated and IL1β-treated chondrocytes. **G**. *DUX4* and **H**. *CABIN1* expression in CHON002 (KO and OE for CD5L) cells either left untreated or treated with 1 μM doxorubicin.

The DUX4 mRNA was upregulated in OA chondrocyte samples relative to non-OA samples (2.7-fold change; *p* = 0.037; Fig. 5A–B). IL1β treatment did not significantly alter *DUX4* expression in primary chondrocytes. Since DUX4 has been implicated in apoptosis initiation, we chose to quantify *DUX4* expression in CHON-002 cells treated with doxorubicin. Intriguingly, we found that *DUX4* expression was repressed in CHON-002 cells in which CD5L had been KO and upregulated in CD5L-overexpressing cells (Fig. 5G).

We observed no significant difference in *LSP1* expression between samples from OA patients and controls. However, we noted a trend toward lower levels of expression in OA samples, suggesting that a larger cohort of patients may be required to assess the significance of changes in *LSP1* expression. In primary chondrocytes treated with IL1β, *LSP1* expression was significantly and markedly downregulated relative to untreated cells (80% decrease in expression, Fig. 5B), with a more pronounced effect in OA samples (95% decrease in expression). Unfortunately, in CHON-002 cell line, *LSP1* expression levels were too low (Ct > 35) for conclusive validation and further analysis.

Consistent with our RNAseq data we found that *CABIN1* expression was increased in CD5L KO cells. Following doxorubicin treatment, CABIN1 expression was reduced in both OE and control samples. We observed considerable variability in *CABIN1* expression between OA samples, with no significant pattern of modulation. Following exposure to 10 ng/μL IL1β for 24 h, both OA and control chondrocytes displayed a similar pattern of significant downregulation of *CABIN1* expression.

## 4 Discussion

Osteoarthritis is a degenerative joint disease characterized by the breakdown of cartilage and the development of chronic inflammation. In recent years, the incidence and number of patients with OA have gradually increased. Current therapeutic options for OA are largely limited to symptom control, primarily through non-surgical pain management. Knee joint replacement surgery is generally reserved for patients with advanced disease who remain severely symptomatic and functionally impaired despite a well-conducted course of conservative treatment in line with clinical guidelines. There is therefore an urgent need to identify new therapeutic targets and develop innovative treatments capable of slowing or halting OA progression.

CD5L is a secreted protein from the scavenger receptor cysteine-rich family. Members of this family are mostly expressed by immune cells, including T cells, B cells, and monocytes, but are also present in non-immune cells where they are thought to play a role in a wide range of metabolic functions [35]. We therefore hypothesized that CD5L might be involved in the development and progression of OA by contributing to inflammation and cartilage degradation in the joints. If our hypothesis is correct, CD5L may stimulate the production of other proinflammatory cytokines and enzymes that can worsen the damage and inflammation occurring in osteoarthritis. In this preliminary study, we investigated, for the first time, the potential roles of CD5L in chondrocytes in OA.

Studies of CD5L in cell culture are challenging because CD5L is present at only low levels in most of cell lines and the protein is not easily detectable by western blotting in WT cell lines. In 1999, Miyazaki et al. had already described the disappearance of the protein from western blots of primary cells after 16 h of culture in a flask [36]. This disappearance of the protein may have several causes, such as instability of the protein during the cell culture process or an increase in secretion. Low levels of CD5L protein pose a challenge in assessing the efficiency of any gene ablation experiment. We overcame this limitation by using CD5L-overexpressing cells to validate the efficiency of our *CD5L* targeting guides.

CD5L protein levels has been shown to be elevated in the SF of patients with osteoarthritis relative to controls [10]. Shoji et al. (2021) also recently reported an increase in CD5L levels in the synovial membranes of obese OA patients [37]. This suggests that CD5L may contribute to synovial inflammation in obese OA patients. SF composition has a direct impact on chondrocyte function and the maintenance of healthy cartilage [38]. In our study, we modeled the increase in CD5L levels in the SF by treating cultured chondrocytes with rCD5L. We found that CD5L could regulate its own expression, leading to an increase in its levels in chondrocytes.

We also treated chondrocytes with IL-1β, which is widely used *in vitro* to model cytokine-driven pro-inflammatory and pro-catabolic signaling [39]. We found that IL1β increases CD5L expression in chondrocyte cell lines and primary chondrocytes from OA and patients. Interestingly, the increase of CD5L expression in non-OA chondrocytes was not significant. The identification of the upstream *in vivo* regulators of CD5L expression in chondrocytes therefore remains an important question for future studies.

For logistical reasons the number of OA samples available to us in this study was small (*n* = 3). This is a limitation of our study, but our observations made in primary cells pave the way for a larger patient-directed study. To mitigate the limitation of our small primary chondrocyte cohort, we analyzed CD5L expression in publicly available bulk RNA-seq data. Because CD5L is expressed at low levels, it was not detectable in many samples. CD5L expression was higher in subgroup A, an inflammatory/immune-related subtype, consistent with its induction by IL-1β in our *in vitro* experiments. These findings highlight the importance of patient endotyping in OA research [40,41], as molecular stratification may be required to detect expression differences masked in unselected cohorts. They also illustrate that low-abundance transcripts such as CD5L can be biologically relevant, and that adequate sequencing depth is critical to avoid their under-detection.

Chondrocyte apoptosis is increased in osteoarthritic cartilage, with TUNEL-based studies reporting roughly a 2–4-fold higher proportion of apoptotic chondrocytes in OA compared with NCs, and this increase has been proposed to contribute to disease progression [42]. This process is a major driver to cartilage degradation [43]. However to date signals initiating induction of apoptosis and chondroptosis (described as a slow apoptosis process [44]) as well as the complete mechanisms underlying these processes in OA remain elusive.

It may be hypothesized that limiting chondrocyte cell death could help preserve cartilage integrity and ECM production, thereby slowing OA progression. This hypothesis is supported by preclinical *in vivo* studies in which inhibition of caspase-dependent apoptosis reduced cartilage degeneration in experimental OA models [45], and by observations that increased chondrocyte death is often associated with more severe matrix damage in animal and human cartilage [46].

Doxorubicin has been reported to be a strong inducer of MMP13, a cartilage-degrading metalloprotease, in primary chondrocytes from OA patients [20], which aligns with our findings. Since MMP13 is commonly used as a marker of chondrocyte apoptosis and matrix degradation and has been shown to be upregulated alongside key apoptotic markers including caspase-3 and cleaved PARP [47], our observation supports that doxorubicin induces apoptotic pathways in chondrocytes. While both MMP9 and MMP13 can respond to pro-apoptotic stimuli, MMP13 may be more sensitive to the specific apoptotic pathways activated by doxorubicin in CHON002 cells, explaining the pronounced upregulation of MMP13 and the modest, non-significant change in MMP9 levels. Beyond metalloproteases, CD5L has emerged as a regulator of apoptosis in myeloid cells [48], with reports of both pro- and anti-apoptotic effects in macrophages [49,50]. Here, we extend pro-apoptotic role of CD5L to chondrocytes. We found that deletion of CD5L conferred protection against doxorubicin-induced apoptosis, whereas CD5L OE did not further enhance cell death. This suggests that the presence of CD5L, rather than its expression level, is critical for apoptosis activation in our experimental context. Overall our findings reveal a novel interplay between CD5L and initiation of apoptosis in chondrocyte cell death.

A key limitation of this work is that experiments that were performed *in vitro* in human cells could not be independently validated in animal models. This is due to substantial differences in regulation of CD5L expression between mice and humans. For example CD5L is highly expressed in mouse macrophages but is produced at very low levels in human macrophages. Moreover, its response to inflammatory stimuli differs between species, suggesting divergent regulation. Specifically Gilbert et al. reported that Cd5l is significantly downregulated (logFC = −1.33, adj. *p* = 2.48 × 10^−5^) in mouse knee cartilage 42 days after DMM surgery [51], whereas we observe CD5L upregulation in human cartilage obtained at the time of knee replacement surgery. Together, these observations indicate that CD5L regulation in murine early OA may not mirror its behavior in human late OA, underscoring the need to develop more complex human model systems (such as ex vivo cartilage explants or joint organoids) to validate CD5L-targeted strategies.

Whole transcriptome RNA sequencing revealed that CD5L OE leads to an increase in *DUX4* expression. These findings were supported by qPCR analysis, which showed that in the absence of CD5L KO doxorubicin treatment fails to induce *DUX4* in the CHON002 cell line. DUX4 is a transcription factor belonging to the homeobox protein family encoded by the *DUX4* gene located in the 4q35 region of human chromosome 4 [52]. *DUX4* expression is tightly regulated and confined to early stages of embryonic development [53]. Aberrant *DUX4* expression has been linked to facioscapulohumeral muscular dystrophy (FSHD), a genetic disorder characterized by progressive muscle weakness and atrophy. *DUX4* misexpression in adult skeletal muscle is believed to contribute to FSHD pathogenesis through various mechanisms, including apoptosis induction, the disruption of myogenesis and the activation of immune and inflammatory pathways. Another study reported that *DUX4* OE induces cell death through the caspase 3/7 pathway in the context of FSHD [54]. To the best of our knowledge, this is the first report of *DUX4* expression in chondrocytes in the context of OA.

*CD5L* OE has been shown to alter the expression of several other genes, potentially leading to further downstream effects on cellular processes. *LSP1* (lymphocyte-specific protein 1) is a gene encoding an intracellular F-actin-binding protein expressed predominantly in hematopoietic cells, particularly leukocytes. The LSP1 protein plays a crucial role in regulating the cytoskeleton [55], cell migration, and adhesion in immune cells, including neutrophils [56], lymphocytes [57], and monocytes [58]. It is involved in various immune cell processes, including leukocyte signaling, motility, and extravasation. Given its importance in immune cell function, LSP1 has been implicated in the regulation of inflammatory responses and has been studied in the context of various inflammatory diseases, including RA [59]. However, its direct role in non-hematopoietic cells, such as chondrocytes, has been less explored.

We observed a modest increase in *LSP1* expression in the CD5L KO cell line, and a substantial downregulation of *LSP1* following IL1β stimulation of primary chondrocytes. These results are consistent with those of Pemmari et al., who demonstrated a downregulation of LSP1 in primary Human chondrocytes by RNA sequencing after IL1β treatment. Intriguingly, this effect was more pronounced in chondrocytes derived from OA patients. Further research is required to determine why OA cells are more susceptible to LSP1 regulation and to assess the impact of LSP1 on chondrocyte metabolism.

Taken together, our RNA-seq data indicate that CD5L alters the expression of multiple apoptosis-related genes in chondrocytes, including DUX4, LSP1 and CABIN1, while previous studies in macrophages and synovial fibroblasts show that CD5L can modulate NF-κB and ERK1/2 signaling and promote cell survival. However, the exact pathways by which CD5L regulates chondrocyte death and inflammatory responses in OA remain to be elucidated.

## 5 Conclusion

In this study, we show that *CD5L* is overexpressed *in vitro* and ex vivo in chondrocytes under OA conditions. The expression of this gene is increased by treatment with IL1β, CD5L, or doxorubicin in chondrocyte cell lines. Our RNA sequencing data showed that *CD5L* OE enhances the expression of genes associated with programmed cell death, whereas CD5L KO decreases the induction of apoptosis driven by doxorubicin. Approaches modulating CD5L are therefore a potential new therapeutic approach for preventing chondrocyte cell death in the joints during OA.

## Declaration of competing interest

The authors declare that they have no competing interest.
