# Supplementary material for: CD5L is a potential negative regulator of chondrocyte apoptosis in osteoarthritis
Source: Osteoarthr Cartil Open. 2026 Jul 4;8(3):100849. doi: 10.1016/j.ocarto.2026.100849 (PMC13400356; doi:10.1016/j.ocarto.2026.100849)
Supplement: Multimedia component 1 [file mmc1.docx]

*Generation of CD5L KO cell lines*:

KO cell lines were selected on puromycin for seven days.  Two CD5L sgRNAs targeting exon 3 of the cDNA sequence were designed with the CRISPOR sgRNA design tool^1^. Oligonucleotides were synthesized (Sigma) and inserted individually into the lentiCRISPRv2-opti vector (#52961, Addgene) according to the oligomer cloning protocol for LentiCRISPRv2. Two targeting clones and non-targeting clone (NT) were validated by sequencing. The following oligonucleotides were used for sgRNA cloning and include cloning overlays for ligation after BsmBI digestion of the lentiCRISPRv2-Opti vector.

|  | Sequence |
| --- | --- |
| Non-targeting sequence | TTTGTAATCGTCGATACCC |
| CD5L guide 1 (KO1) | CCTCCACCGCTGTGAAGGGC |
| CD5L guide 2 (KO2) | CGGCTGGGACATTAAGGACG |

**Table 1:** List of sgRNA sequences

Lentiviruses were generated as previously described^2^. In brief, 2×10^5^ HEK 293T cells were used to seed a six-well plate. On the following day, the cells were transfected with 1 μg of the psPAX2 plasmid (#12260, Addgene), 0.67 μg of the pMD2.g envelope plasmid (#12259, Addgene) and 1.33 μg of the lentiCRISPRv2-opti sgRNA plasmids (with the NT sg sequence or the CD5L sg sequence), in the presence of the GeneJuice transfection reagent (3 to 1 ratio, 70967, Merck). The medium was changed 12 hours post-transfection. The virus-containing supernatant was collected on day 3 and stored at -20 °C for a short period of time.

We transduced 1×10^6^ CHON002 or TC28A2 cells in 0.5 mL medium with 1.5 mL virus-containing supernatant in the presence of 6 μg/mL polybrene (Sigma Aldrich - TR-1003-G). The cells were incubated overnight and then resuspended in fresh medium, with 2 μg/mL puromycin (Thermo Fisher Scientific - A1113803) added 24 h after transduction. In chondrocyte basal levels of CD5L are too low for direct assessments of KO efficiency by western blotting. We therefore confirmed the efficiency of KO guides targeting CD5L by western blotting after the overexpressing of CD5L in the CHON002 cell line and CRISPR Cas KO.

*Reverse transcription and Quantitative PCR:*

| **TARGET GENE** | **FW SEQUENCE (5’-3’)** | **REV SEQUENCE (5’-3’)** |
| --- | --- | --- |
| GAPDH | GGAGCGAGATCC CTCCAAAAT | GGCTGTTGTCATACTTCTCATGG |
| CD5L | GACGAGAAGCAACCCTTCAG | CCCAGAGCAGAGGTTGTCTC |
| CABIN1 | ATCCTCACTGTGAAGGTGCTCGAA | TTTGGTCTGCACTGTCTCCTGCAT |
| LSP1 | AGGACCGAGTCCCTAAACCG | CTGGGTGTATTGTTCCAGCCA |
| DUX4 | CAGAATGAGAGGTCACGCCA | CCTTCTCAAAGGCTCGGAGG |

**Table 2**: Primers used for quantitative PCR in this study.

*immunofluorescence*:

| Antibody / Stain | Species | Dilution | Secondary antibody incubation time and temperature |
| --- | --- | --- | --- |
| DAPI | - | 300 nM |  |
| CD5L F1, Santa Cruz Biotech, sc-514283 | mouse | WB: 1/1000  IF: 1/200  IHC: 1/200 | 1 h room temperature |
| Invitrogen #A32766) | Monkey  Anti-mouse | IF:1/1000 | 1 h room temperature |
| HRP #P0447 Dako | Rabbit anti-mouse | WB: 1/5000 | 1 h room temperature |

**Table 3:** Reagents and antibodies used for western blotting (WB), immunofluorescence (IF) and immunochemistry (IHC).

Annexin V apoptosis and necrosis assays

Kinetic assay. The RealTime-Glo Annexin V Apoptosis and Necrosis Assay (Promega) was performed according to the manufacturer's instructions. Cells were seeded in white 96-well plates with a clear bottom at a density of 10,000 cells per well and allowed to adhere overnight. Cells were then treated with 1 µM doxorubicin in the presence of the RealTime-Glo detection reagents. Luminescence (apoptosis) and fluorescence (necrosis) signals were recorded every 15 minutes over a 15-hour time course at 37°C using an Infinite M Plex microplate reader (Tecan).

Endpoint fluorescence microscopy. Cells were seeded in chambered cell culture slides (IBIDI, 8-well, #80841) and treated with 1 µM doxorubicin. At selected time points, cells were washed with cold PBS and stained using the Annexin V-FLUOS Staining Kit (Roche) according to the manufacturer's instructions. Annexin V-FLUOS conjugate was diluted in binding buffer, added to the cells, and incubated for 15 minutes at room temperature in the dark. Following three washes, cells were imaged by fluorescence microscopy (EVOS FL Auto). The percentage of Annexin V-positive cells was quantified for each condition.

References for supplementary material

1. Concordet JP, Haeussler M. CRISPOR: intuitive guide selection for CRISPR/Cas9 genome editing experiments and screens. *Nucleic Acids Res*. 2018;46(W1):W242-W245. doi:10.1093/nar/gky354

2. Nejati Moharrami N, Bjørkøy Tande E, Ryan L, Espevik T, Boyartchuk V. RORα controls inflammatory state of human macrophages. *PLoS One*. 2018;13(11):e0207374. doi:10.1371/journal.pone.0207374

3. Pattappa G, Karlsson NG, Steinecker-Frohnwieser B, et al. Towards stratification in osteoarthritis: a review of the scientific terminology used in published basic research. *BMC Rheumatol*. 2025;9:109. doi:10.1186/s41927-025-00557-0
