# Supplementary material for: CD5L is a potential negative regulator of chondrocyte apoptosis in osteoarthritis
Source: Osteoarthr Cartil Open. 2026 Jul 4;8(3):100849. doi: 10.1016/j.ocarto.2026.100849 (PMC13400356; doi:10.1016/j.ocarto.2026.100849)
Supplement: Multimedia component 2 [file mmc2.docx]

**Supplementary Figure 1**
Safranin-O staining (top panels) and CD5L immunohistochemistry (bottom panels) in two independent human osteoarthritic cartilage samples (DW0960 and DW130A). Sections DW0960 and DW130A are from distinct donors with mild OA (Mankin score ≤3) and were used solely to confirm CD5L protein localization in and around chondrocytes; they are not matched to the qPCR cohort. Safranin-O highlights proteoglycan content, while brown DAB signal indicates CD5L distribution. Scale bars: 100 µm.


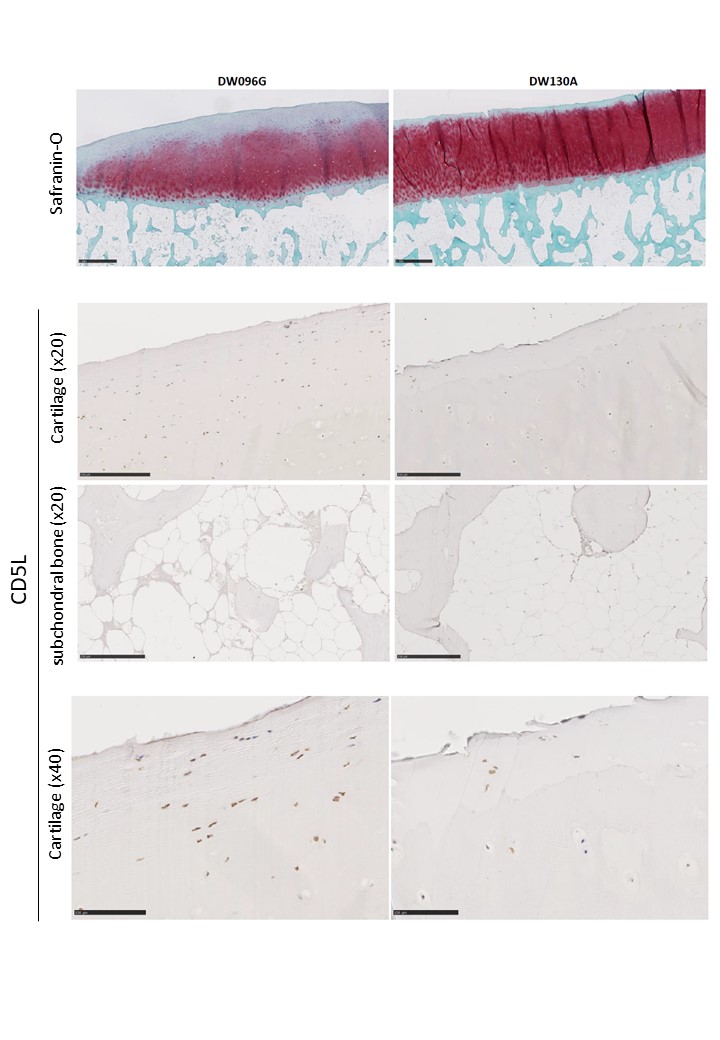


**Supplementary Figure 2:**

CD5L transcript levels in human knee cartilage from OA and non-OA patients, derived from publicly available bulk RNA-seq data (GSE114007).


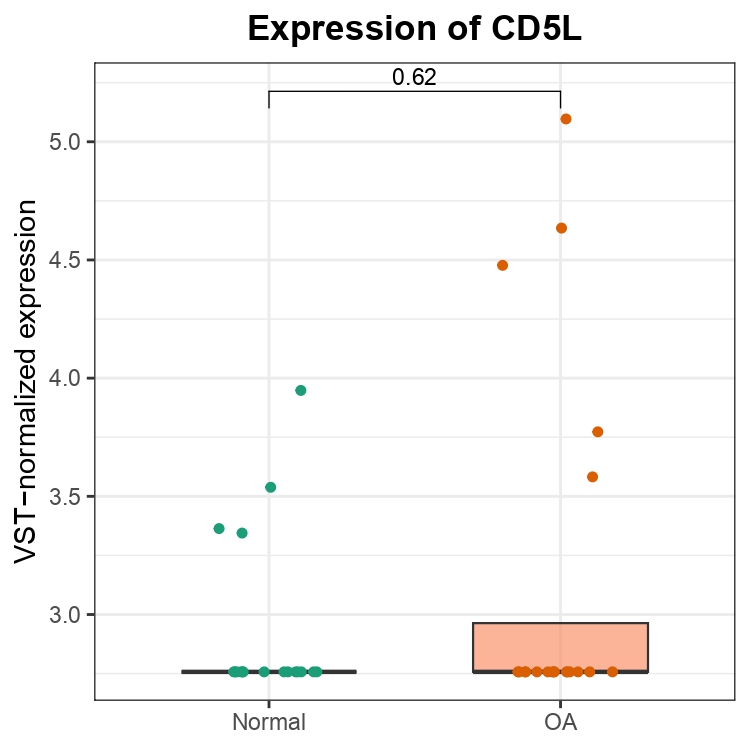


**Supplementary Figure 3:**

*CD5L* gene expression in CHON002 cells treated with sodium iodoacetate for 24 h.


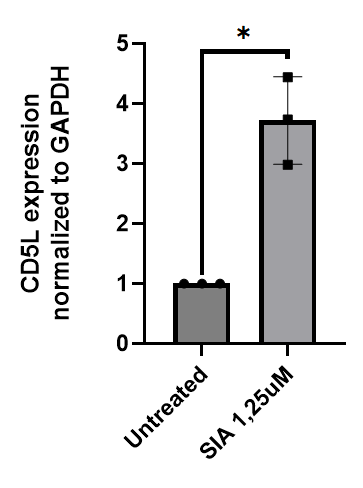


**Supplementary Figure 4**

Cell viability of TC28A2 cells was measured using the CellTiter-Glo Luminescent Assay under control conditions (A) and following 24 h treatment with 1 µM doxorubicin (B).


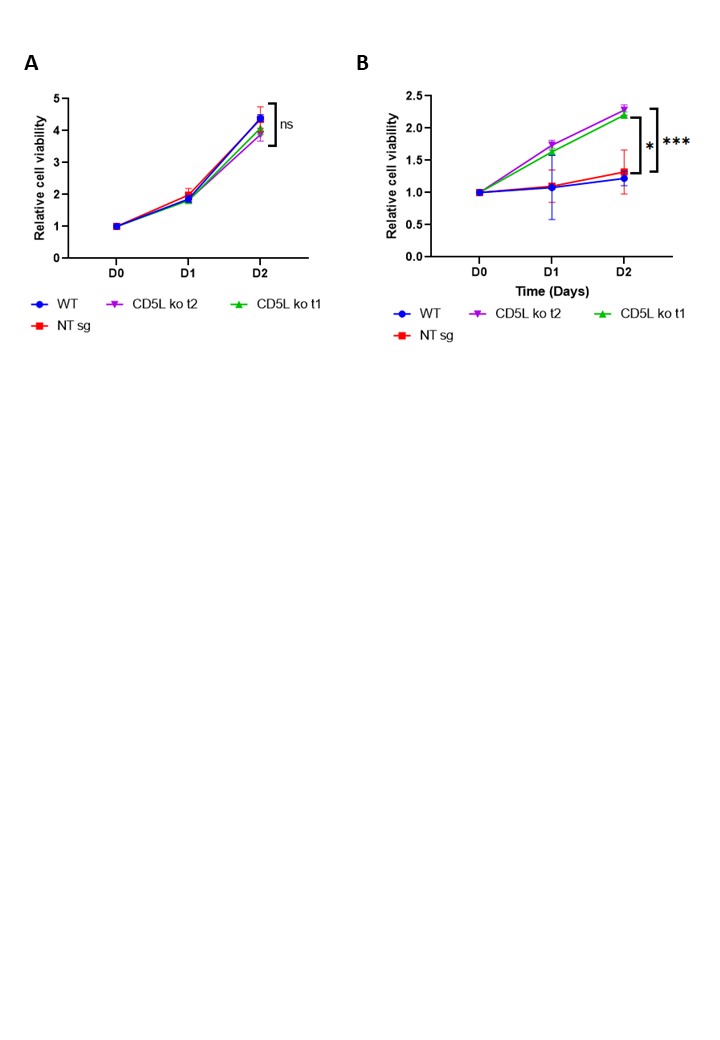


**Supplementary Figure 5**

RealTime-Glo necrosis assay showing fluorescence (RFU) over 15 h in CHON-002 cells treated with 1 µM doxorubicin, reflecting the time-dependent activation of necrosis.

**
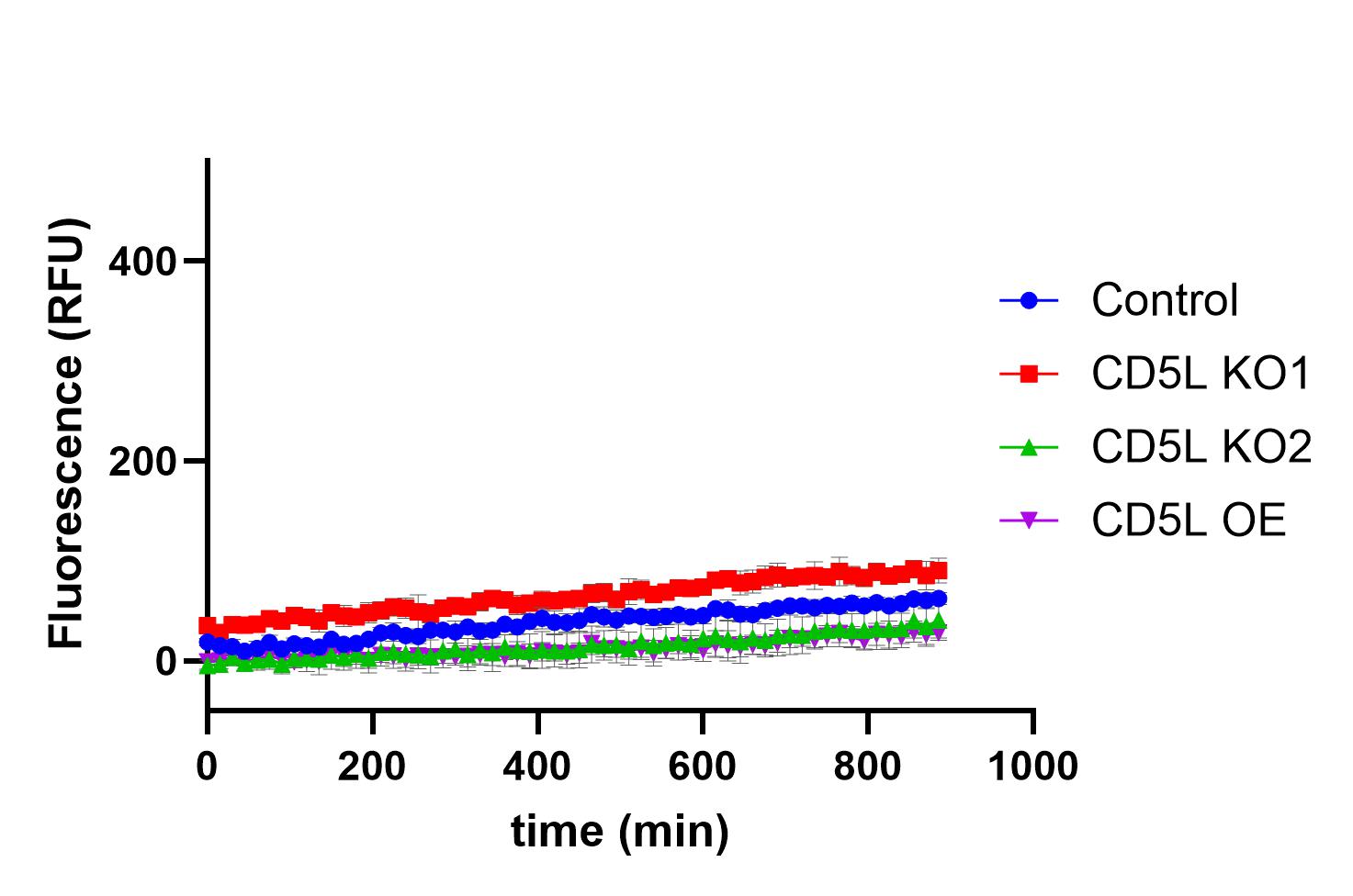
**

**Supplementary Figure 6**

1. Annexin V staining after 24 h doxorubicin treatment in the CHON002 cell line and associated quantification (**B**). Error bars indicate the mean ± SD of at least four independent experiments. ns non-significant, ***P* < 0.01; ****P < 0.0001. **
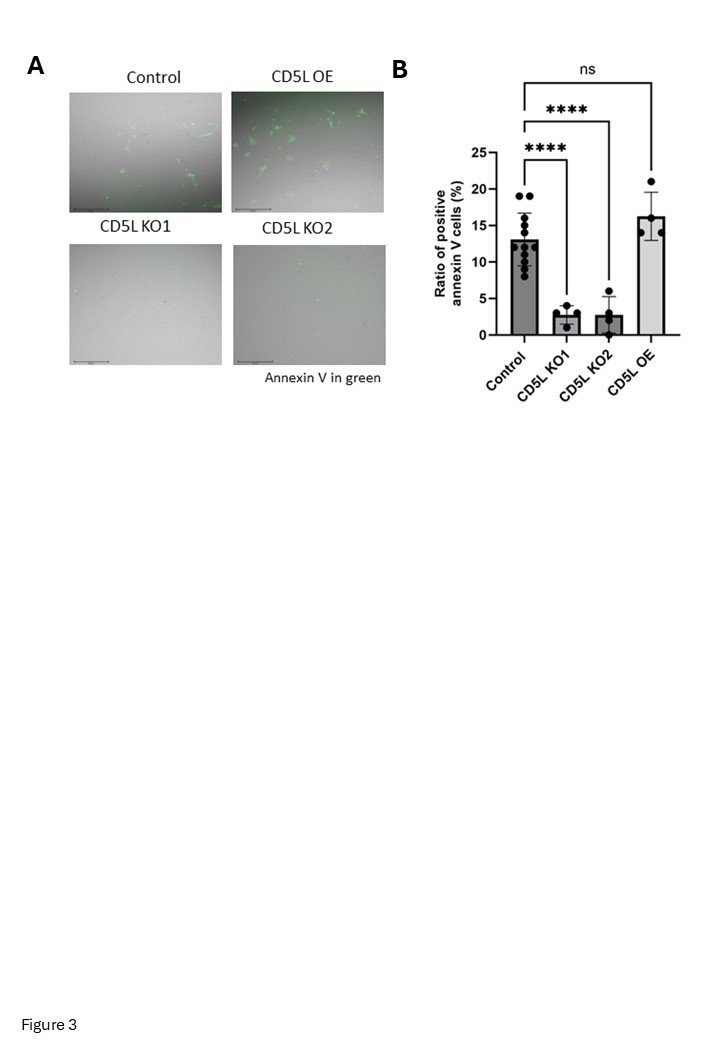
**
